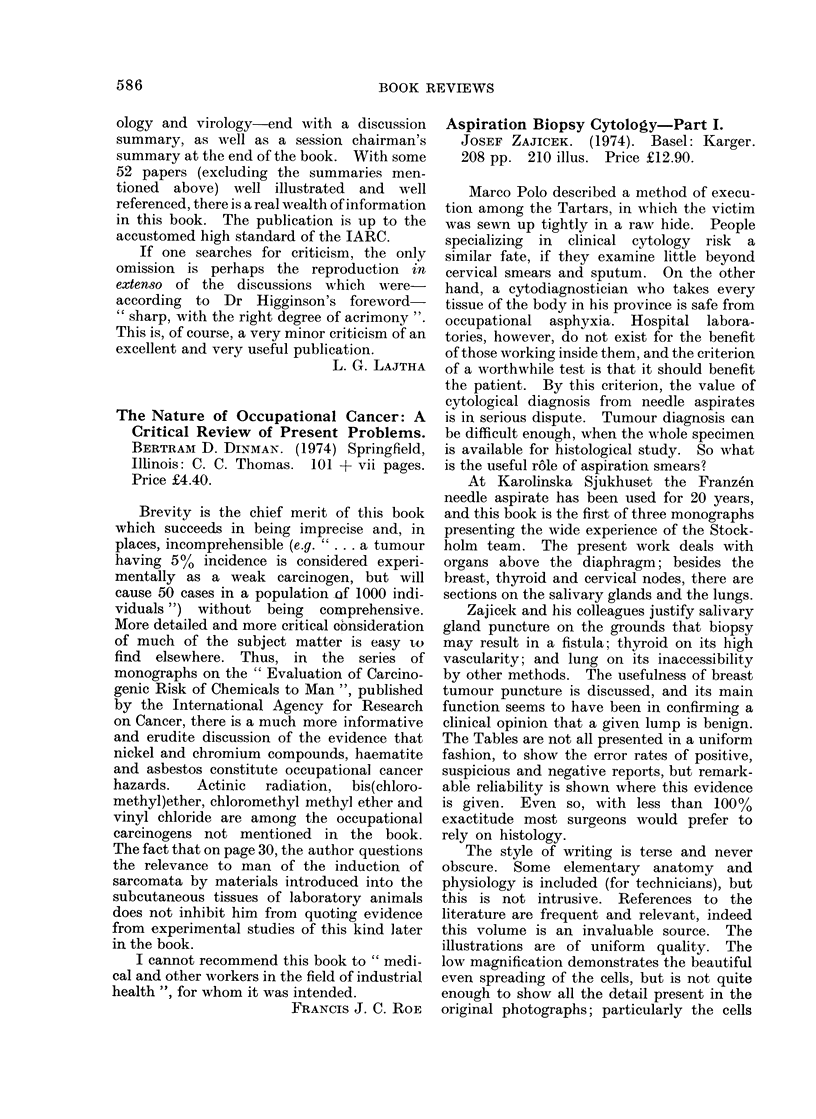# The Nature of Occupational Cancer: A Critical Review of Present Problems

**Published:** 1974-12

**Authors:** Francis J. C. Roe


**The Nature of Occupational Cancer: A Critical Review of Present Problems.** BERTRAM D. DINMAN. (1974) Springfield, Illinois: C. C. Thomas. 101 + vii pages. Price £4.40.

Brevity is the chief merit of this book which succeeds in being imprecise and, in places, incomprehensible (*e.g.* " . . . a tumour having 5% incidence is considered experimentally as a weak carcinogen, but will cause 50 cases in a population of 1000 individuals ") without being comprehensive. More detailed and more critical consideration of much of the subject matter is easy to find elsewhere. Thus, in the series of monographs on the " Evaluation of Carcinogenic Risk of Chemicals to Man ", published by the International Agency for Research on Cancer, there is a much more informative and erudite discussion of the evidence that nickel and chromium compounds, haematite and asbestos constitute occupational cancer hazards. Actinic radiation, bis(chloromethyl)ether, chloromethyl methyl ether and vinyl chloride are among the occupational carcinogens not mentioned in the book. The fact that on page 30, the author questions the relevance to man of the induction of sarcomata by materials introduced into the subcutaneous tissues of laboratory animals does not inhibit him from quoting evidence from experimental studies of this kind later in the book.

I cannot recommend this book to " medical and other workers in the field of industrial health ", for whom it was intended.

FRANCIS J. C. ROE